# Clinical Remarks upon Surgical Cases in the Buffalo General Hospital—Excision of the Globe of the Eye—Operation for Cataract—Radical Cure of Varicose Veins

**Published:** 1864-01

**Authors:** J. F. Miner


ART. III.—*Clinical Remarks upon Surgical Cases in the Buffalo General Hospital—Excision of the Globe of the Eye—Operation for Cataract—Radical Cure of Varicose Veins.* By J. F. Miner, M. D.

November 12, 1863.

Gentlemen:—I should perhaps preface my remarks this morning by a brief account of the progress which the patients, operated upon last week, are making towards recovery. The young lady whose thigh we amputated has not apparently suffered in the least from the operation; she has eaten, slept and felt as well as ever; the wound has united by what is called "first intention," and the only opening now present is that kept up by the ligatures. It is a most remarkable result in many respects, and one that was by no means anticipated. How a young lady could have amputation of the thigh, and not suffer either shock, pain, loss of appetite, sleep, or anything else common after such operations I am unable to explain; such, however, has been the fortunate result.

*The 2d Case*—Removal of Tumor—has also been equally successful, and I am happy to inform you, that the old gentleman, now 70 years of age, is apparently the happiest man in the world.

The first case we present before you this morning is, Cataract—opacity of the crystaline lens—a very common disease with old people, and not unfrequently congenital—existing at birth. This case is one of great interest to me, not so much on account of the eye which has the opake lens still remaining, but the other eye where the lens has been removed. The left eye has been operated upon by a very skillful and accomplished surgeon with perfect success, so far as removal of the cataract is concerned; but vision is not restored. The humours of the eye are perfectly transparent—and so far as appearances indicate there should be good vision; yet he is blind.

Many of you are perhaps familiar with the object and uses of the Ophthalmoscope—an instrument by which we are able to light up and look into the posterior chamber of the eye, to see some of the changes which disease produces upon the delicate tissues of the organ of vision. It is remarkable with what distinctness some of these changes are observed; though it must be confessed that blindness may be complete, and the causes yet remain undiscovered. If you have never become familiar with the workings of the ophthalmoscope, your enthusiasm will be raised to the highest point when you are able to catch your first glimpses of its wonderful revelations. It seems as though you had borrowed the eye of

Omnipotence and was looking in upon the secret workings of one of the most remarkable organs of physical machinery, whose hitherto obscure chambers are now lighted up by the torch of science. I am not sufficiently familiar with the use of this instrument to be able to see with distinctness all the conditions which are described by authors, but I think I am able to see the reason why this patient does not enjoy good vision. I think I can see that the retina has suffered change, and suppose that loss of sight depends mainly upon this delicate membrane being unfitted to receive impressions.

I have no reason to suppose that this was in any way produced by the kind or mode of operation practiced upon the eye. The changes required to destroy vision are very slight, and are liable to occur after any, and all operations for cataract. I shall make upon the other eye the operation by division or depression or both, and hope for better results, though I cannot be sure of obtaining them.

The process of absorption will gradually remove the lens after the breaking up of the capsule and depression of lens, which you now observe the process of making. It is quite painless and bloodless, and is generally followed by good results. The operation by extraction is now generally regarded as most likely to be followed by permanent vision, and many of the best operators greatly prefer this method, as being less liable to be followed by inflammation and the condition which is present in the left eye of this patient. I have practiced both methods of operation, and am not yet prepared to express personal opinion as to their relative advantages. I have obtained good results by extraction, depression and division, and I haye also had cases of failure. This patient has come from Niagara county for this operation, and will remain sufficiently long to enable us to judge something of the success of our efforts to restore vision.

*Case 2d.*—This young man, now 20 years of age, received when quite a boy, a severe blow upon the eye, by which vision was immediately destroyed. The eye has somewhat the appearance of melanosis—that is, the iris is changed, appearing black, and without any central opening or pupil. I have previously satisfied myself more fully of the condition of this eye, by introducing a hook into the anterior chamber, and attempting to draw out the iris, as in artificial pupil; but I find that there is no iris, and that its place is supplied by the products of inflammation. This condition has produced so great intolerance of light in the sound eye, that he has not been able to follow useful employment, but a very small part of his life; he is never able to look up in good light, without shading the eye.

The sympathy which exists between these organs is very remarkable, while the exact conditions upon which it depends are not very thoroughly understood; the principles of treatment, however, which are proper in such cases, are better defined; especially for the last few years has it been fully established, that medical treatment exerts but little if any favorable influence in severe cases over sympathetic inflammation of the eye, after injury, and that excision or extirpation constitutes the only remedial procedure from which we can expect any decided benefit; while this affords prompt and permanent relief.

With the expectation of relieving this young man of the sympathetic effects of this diseased eye, I shall excise the anterior portion of the globe, and allow the escape of the contents of the eye, which you now observe consists of a fibrinous clot in place of the iris, and serum in place of the vitreous humor. Simple dressings will be applied, and I shall hereafter report to you the results of the operation. If it should prove as successful as I expect, he will have perfect vision in the healthy eye, and a good stump upon which to rest an artificial eye in place of the one we have excised. Nothing can be lost by making this operation, since he had no vision in the diseased eye, and I have confident expectation that much will be gained every way—relief of the intolerance of light—protection from injurious inflammation, and place for artificial eye—this I am expecting from the operation; but you are well aware there is uncertainty of result in all operations of this nature.

You observed that after removing the anterior portion of the globe the contents of the eye did not escape, and that the forceps removed with some effort the fibrinous clot which was adherent to the sclerotic coat and retained in the posterior chamber an accumulation of yellow serum, which had taken the place of the vitreous humour. This disorganization is the result of inflammation, and must be a rare product in this position, even of long continued inflammatory action. The contents of the orbit will gradually contract as the process of granulation progresses, and the young man will probably be relieved of a malady which has embittered his whole life thus far, and made him at times intensely miserable.

*Case 3d.*—The third and last operation we shall make this morning is for the radical cure of *varicose veins.* Surgeons practice several different operations for this purpose, all having the same object—obstruction of the vessel. Ligation of the vessel is perhaps the more common. Destruction by caustics, in various forms, has also been practiced. This patient has had, I judge, caustic potassa applied with this view of exciting suppurative inflammation in the integument and vessel, and of thus closing and preventing the circulation of blood, forcing it to deeper and undilated channels, but has failed in every respect. This large, indolent ulcer has been here for several years, and some of his physicians have even regarded it as indicative of caries of the tibia, and proposed operation for removal of diseased bone. I need not say a more erroneous view could not have been entertained. The condition of the circulation is the cause of this ulcer, and when we have obliterated these immensely dilated veins, we shall soon see the ulcer take on healthy granulation and disappear, while the bones of the leg have never been in the least diseased.

Within the last few years a somewhat new method of operation has been practiced by a few surgeons—injection of solution of persulphate of iron into the vessel, with the view of coagulating the blood and thus causing a complete stoppage of the circulation. I have adopted this operation, practicing it in a great number of cases, both in private and hospital practice, and have never seen any unpleasant or unfavorable results; it has been with me uniformly safe and successful. I claim to speak only for myself, and would not hold any one else responsible for my opinions, since I am aware that others entertain different views concerning the safety and propriety of this operation. I believe that I am the only surgeon in this city who has ever adopted this plan, giving it a fair trial. It has been attempted by others, but from some cause, either abandoned or completed by application of ligatures. I have my own way of explaining this want of success, and do not regard it as any reflection upon the efficiency and safety of this operation.

I now take a small scalpel and open down, exposing the vessel, which is at once distinguished by the color given it, by the dark venous blood. With hypodermic syringe I inject into the vessel solution persulphate of iron four drops, diluted with about forty drops of water. This causes an instantaneous coagulation of all the blood in the vessel between the two points of compression, which may be made in any way you please; the fingers of an assistant answer every purpose. The clot should not be made more than an inch in length. It may be made in one or two different places in the same vessel; all the larger vessels should be treated in this manner.

The details of this operation are of importance; a faithful observance of these will insure success, Opening down through the integument will prevent any uncertainty as to where you are to dislodge the contents of the syringe; thorough compression of the vessel will limit the extent of your clot; careful preparation of your solution will guard against its being used in so concentrated form as to destroy the coats of the vessel and produce extensive slough; application of large, warm poultice will abate the smarting and pain, and guard against inflammation. Hoping the results of this operation—of which I will inform you—will prove as favorable as my description has led you to expect, I invite you to adopt it, when opportnnity offers, and judge for yourselves its relative merits.

